# Indications and outcomes of keratoplasty ≤ 5.5 mm diameter (“mini-keratoplasty”)

**DOI:** 10.1186/s12886-023-03150-6

**Published:** 2023-10-10

**Authors:** Hila Fathai, Gerd Geerling, Johannes Menzel-Severing

**Affiliations:** Department of Ophthalmology, Heinrich Heine University Düsseldorf, Düsseldorf, Germany

**Keywords:** Corneal transplantation, Corneal ulcer, Keratoplasty, Corneal graft, Corneal perforation

## Abstract

**Purpose:**

To report indications and clinical outcomes of corneal grafts ≤ 5.5 mm in diameter (“mini-KP”) in a German tertiary referral center.

**Methods:**

Patients who had undergone mini-KP to treat corneal ulcers with or without perforation between 2011 and 2018 at the Department of Ophthalmology, University of Düsseldorf, Germany, were identified from the local keratoplasty registry. All patient records were reviewed for age, gender, laterality, systemic and ophthalmological diseases, etiology of the corneal ulcerative disease, pre- and postoperative visual acuity over a follow-up time of up to 12 months, graft size, postoperative complications and the need for and timing of further corneal interventions.

**Results:**

37 eyes of 37 patients (male: n = 20; female: n = 17) with a mean age (± standard deviation) at presentation of 70 ± 18.8 years (range: 22–92 years) were identified. Most common etiologies were neurotrophic keratopathy (n = 15), dysfunctional tear syndrome (n = 9) and atopic keratoconjunctivitis (9). Mean graft diameter was 4.51 ± 0.63 mm (range: 3-5.5 mm). 23/37 eyes (62%) required no further intervention in the acute phase. 14/37 patients (38%) required secondary corneal intervention, due to complications. One-year graft survival was 78.4%. One eye had to be eviscerated due to recurrent corneal ulceration and endophthalmitis. 36 of 37 eyes were preserved. We found a highly significant correlation between type 2 diabetes and the development of postoperative complications (r = .46; p = .005). Corrected distance visual acuity (CDVA) improved from 1.42 ± 0.75 logMAR to 0.9 ± 0.65 logMAR postoperatively (t (23) = 5.76; p < .001).

**Conclusion:**

Mini-KP can be used successfully in eyes with advanced corneal ulcers due to various infectious and noninfectious etiologies to restore tectonic stability in the long-term and with moderate visual gains.

## Introduction

Corneal perforation is an ophthalmological emergency, which is often caused by various types of infectious and noninfectiousdisorders, such as infectious keratitis, trauma or autoimmune disease [1]. Untreated, it can lead to severe loss of visual function, secondary glaucoma, endophthalmitis or, in the worst case, to blindness and loss of the eye [2]. Surgical and/or non-surgical treatment is required to rebuild the anatomic integrity of the globe, to preserve vision and to reduce the risk of complications.

The choice of treatment in corneal ulcers is determined by size and location of the defect as well as etiology and immune status of the patient [3]. Nonsurgical treatments such as bandage contact lens and cyanoacrylate tissue glue can be helpful especially in small corneal perforations less than 2.0 mm in size. They can stabilize the eye, avoiding the need for surgical modalities such as amniotic membrane transplantation or conjunctival flap [4], [5]. However, in perforations larger than 3.0 mm in diameter a penetrating keratoplasty is recommended [3].

**Fig. 1:**
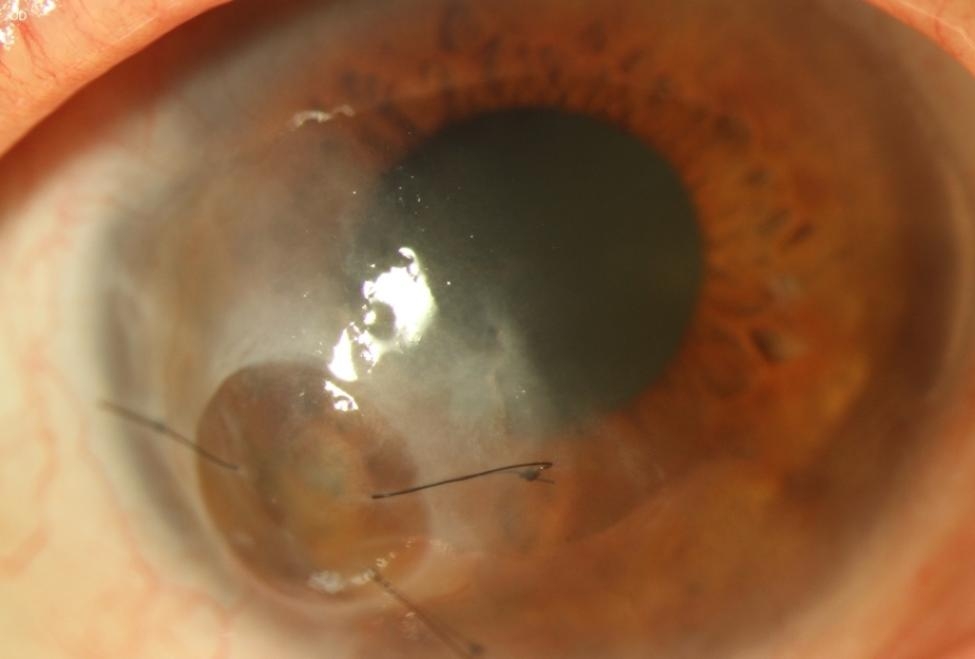
Representative image post mini-keratoplasty of a male, 70-year-old patient treated with a 3 mm size graft for a perforated ulcer in the right eye, due to neurotrophic keratopathy, atopic keratoconjunctivitis and dysfunctional tear syndrome; systemic/ophthalmological diseases: diabetes mellitus type 2, atopic dermatitis, severe keratoconjunctivitis sicca, keratoconus; CDVA in logMAR: 0.8 (pre-operatively); 1.0 (6 and 12 months post-operatively); An AMT-Onlay was performed one week post mini-KP due to a recurrent corneal erosion

“Mini-keratoplasty” (mini-KP) is a corneal grafting technique that uses a small graft, which is frequently placed outside the visual axis to save the eye and potentially avoid sustained loss of vision (Fig. 1). The first successful attempt of small-diameter, round and eccentric tectonic keratoplasties were reported by Ascher in 1919, followed more recently by Hallermann in 1972 [6], to treat peripheral ulcerative corneal disorders [7], [8]. Hallermann defined mini-keratoplasty as the transplantation of a graft with a diameter of 2-5 mm [9]. In principle, however, every diameter that spares the corneal center and thus the area of the visual axis, when placed as an eccentric graft, can be considered a “mini-KP”. In this retrospective case series we report indications and clinical outcomes of corneal grafts ≤ 5.5 mm in a German tertiary referral center.

## Methods

We conducted a comprehensive, retrospective search of hospital records to identify all patients in which a mini-KP had been performed to treat a corneal ulcer with or without corneal perforation between 2011 and 2018 at the Department of Ophthalmology, University of Düsseldorf, Germany. Mini-KP was defined as penetrating graft with a maximum diameter of 5.5 mm. This was the only inclusion criterion; exclusion criteria were not defined. The surgical technique consisted of manual trephination of the graft and host bed. In the recipient, trephination was centered on the lesion and the graft was sutured in place using nylon 10 − 0 single sutures. Postoperative standard therapy consisted of unpreserved topical antibiotics 4 times a day, topical dexamethasone starting at 5 times a day (reduced monthly by one drop), gentamicin and dexamethasone ointment at night, and frequent unpreserved lubricant eye drops. Patients with a history of viral pathogenesis in addition received topical and - if required - systemic acyclovir 5 times a day. Suture removal was aimed for at one year post-surgery.

Patient records were reviewed for age, gender, laterality, systemic and ophthalmological diseases, etiology of the corneal perforation or ulcer, pre- and postoperative visual acuity within a follow-up time of up to 12 months, graft size, postoperative complications, graft failure and the necessity and timing of further corneal intervention.

The etiologies which lead to mini-KP were categorized as neurotrophic keratopathy, dysfunctional tear syndrome, atopic keratoconjunctivitis, active herpetic keratitis, rheumatic disease, or trauma.

“Anatomic success” was defined as no need for further corneal intervention within 12 months after initial mini-KP. The need for secondary minor corneal procedures such as amniotic membrane transplantation (AMT) or cyanoacrylate glue was defined as “qualified anatomic success”. A second mini-KP, a larger penetrating keratoplasty or evisceration during the 12-month follow-up period were designated as “anatomic failure”.

Visual acuity had been obtained using Snellen charts, was converted to logMAR for statistical analysis and considered as “full functional success” (i.e. ability to read) with a corrected distance visual acuity (CDVA) of 0.4 dec or 0.4 logMAR. A postoperative visual acuity of 0.05 dec (1.3logMAR) or better, which is relevant for outdoor orientation, was designated as “qualified functional success”. CDVA worse than 1.3 logMAR was defined as “functional failure”. One patient did not have any follow up visual acuity data and therefore was excluded from the CDVA analysis.

Statistical analysis was performed using SPSS software for Windows, version 25 (SPSS Inc., Chicago, IL, USA). All values are presented as mean ± standard deviation. Baseline characteristics and clinical outcome data were compared between groups or times during the 12-month follow-up by using the student’s t-test for unpaired or paired groups. A p-value of < 0.05 was considered statistically significant. Correlation coefficients for the occurrence of certain diseases and development of postoperative complications within the “mini-KP” cohort were calculated by Pearson-Correlation in SPSS.

This study adheres to the principles outlined in the Declaration of Helsinki and to German federal and state laws. The study received ethical approval by the institutional review board (study number 2019 − 677).

## Results

### Baseline demographics and clinical characteristics of the patient cohort

**Fig. 2:**
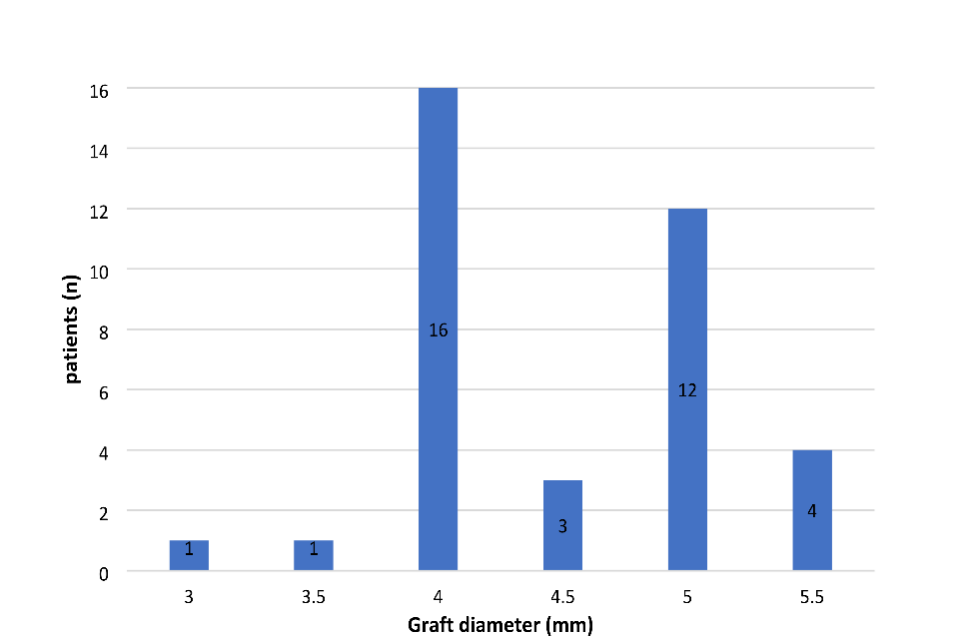
Graft diameter in mini-KP patients

37 patients with mini-KP, 20 male and 17 female, with a mean age (± standard deviation) at presentation of 70 ± 18.8 years (range: 22-92years) were identified with no difference of age between the 2 sexes (men 66.1 ± 20.41 years, women 75 ± 16 years, t (35) = -1.48; p = .148). 19 patients underwent mini-KP in the right and 18 in the left eye. Concomitant systemic conditions are shown in Table 1. Neurological diseases in our cohort were: Alzheimer’s disease n = 1, stroke n = 1, neuro-Behcet’s disease n = 1, polyneuropathy n = 2, facial paralysis n = 1 and abducens nerve palsy n = 1. Overall, 34/37 (92%) had known ocular comorbidities, such as cataract (n = 11), glaucoma (n = 4), are-related macular degeneration (n = 2) or keratoconus (n = 1). The most common etiology for mini-keratoplasty was neurotrophic keratopathy (n = 11), followed by dysfunctional tear syndrome (consisting of: Graft versus host disease n = 1, Steven-Johnson-syndrome n = 1, ocular pemphigoid n = 1, facial nerve palsy n = 1, abducens nerve palsy n = 1, severe keratoconjunctivitis sicca n = 4; Table 2). 28 of 37 eyes (76%) were perforated. Trephination size (Fig. 2) ranged from 3 to 5 mm and mean graft diameter was 4.51 ± 0.63 mm (range: 3.0-5.5 mm).

**Table 1:** Systemic diseases and correlation with post-operative complications

Systemic disease	n*	r (correlation coefficient)	p (significance)
Hypertension Diabetes mellitus Cardiac Dermatological Neurological Rheumatic Pulmonary	18 11 10 9 7 5 4	0.244 0.46 0.24 0.066 0.255 0.009 0.172	0.152 0.005 0.158 0.703 0.134 0.958 0.316

*Due to multimorbidity, several systemic diseases were found in some patients. Therefore, the list of systemic diseases adds up to more than 37

**Table 2:** Etiology of corneal ulcer/perforation (n = 37)

Etiology	n*
Neurotrophic keratopathy	16
Dysfunctional tear syndrome	9
Atopic keratoconjunctivitis	9
Active herpetic keratitis	7
Rheumatic ulcer	5
Trauma	4

*Due to multimorbidity, several possible etiologies were found in some patients. Therefore, the list of possible etiologies adds up to more than 37

### Anatomic outcome

23/37 eyes (62%) did not require any further surgical treatment of the cornea, meaning that full anatomic success was achieved. Among those were all seven patients with active herpetic keratitis. A significant correlation was found between active herpetic keratitis as etiology for corneal disorder and full anatomic success (r = .39; p = .018). Qualified anatomic success, defined as the need of secondary minor corneal intervention but not a second graft, was found in 6/37 (16%) and anatomic failure in 8/37 (22%) of all eyes. Three out of these eight patients required a second mini-KP and five patients required secondary PKP. One-year graft survival therefore was 78.4%. The causes of graft failure were endothelial failure (62.5%) and immunologic endothelial rejection (37.5%).

### Functional outcome

**Fig. 3:**
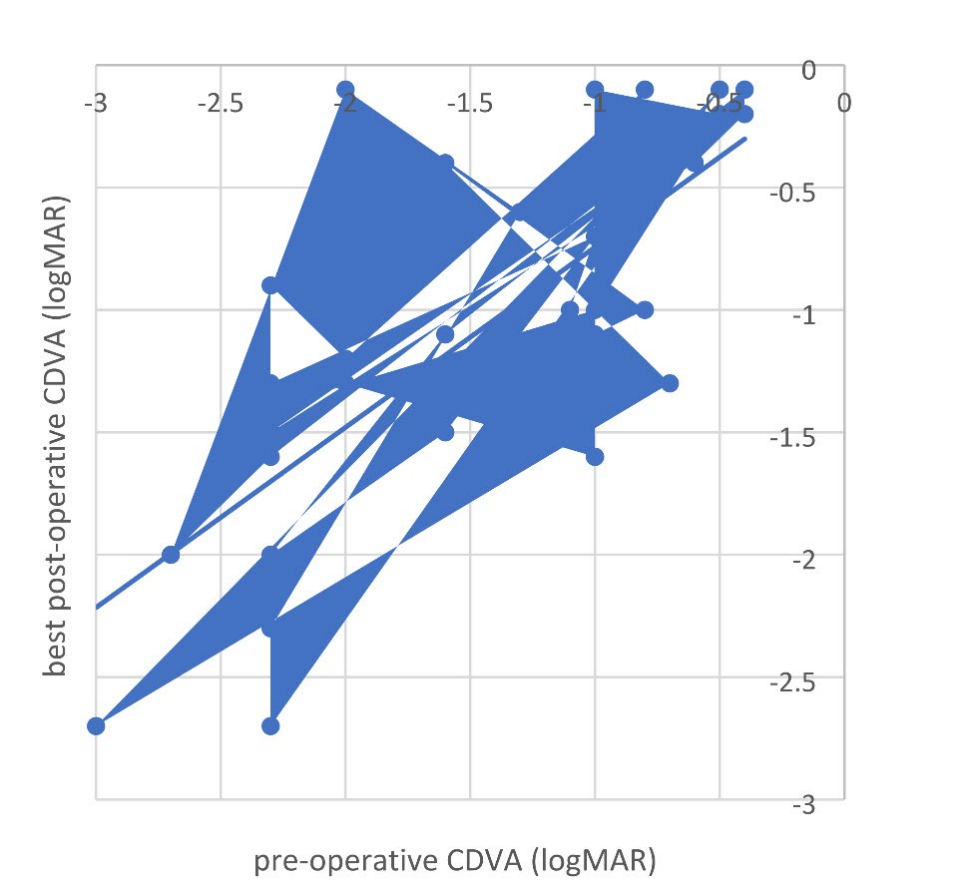
Pre-operative CDVA and best post-operative CDVA (achieved after a mean follow-up of 7.33 ± 8.23 months after mini-KP). There is a statistically significant improvement of visual acuity within the cohort after mini-KP (t (23) = 5.76; p < .001)

Mean (± standard deviation) CDVA was 1.42 ± 0.75 logMAR before mini-KP, 1.7 ± 0.54 one day after mini-KP, 1.5 ± 0.72 at 6 months postoperatively and 1.3 ± 0.86 at 12 months postoperatively. The best postoperative CDVA of 0.9 ± 0.65 logMAR was achieved on average at 7.33 ± 8.23 months after mini-KP. At 6 months follow-up, 30% of the patients had improved visual acuity, while 17% were stable, and in 53% vision had deteriorated compared to preoperatively. At 12 months postoperatively, CDVA had improved in 37.5%, remained stable in 17% and deteriorated in 55.5%. A significant improvement was found between mean preoperative visual acuity of 1.42 ± 0.75 logMAR and best postoperative acuity of 0.9 ± 0.65 logMAR (t (23) = 5.76; p < .001; Fig. 3), with no difference between 6 and 12 months. At 12 months, full functional success was achieved in 10 of 36 eyes (28%), qualified functional in 13 of 36 patients (36%), while the result in 13 eyes (36%) was categorized as functional failure.

### Complications

14/37 eyes (38%) developed postoperative complications in the first year after mini-KP. Of these 14 eyes, 10 were re-operated within the first 4 weeks after mini-KP: Five underwent AMT, one was treated with cyanoacrylate glue, two underwent repeat mini-KP, and six patients required secondary full-size penetrating keratoplasty after initial mini-KP. One evisceration was necessary because of recurrent corneal ulceration and endophthalmitis after repeat keratoplasty using a standard (full-size) graft, in a 49-year-old female patient with a blind phthitic eye, a history of retinopathy of prematurity and multiple intraocular surgeries. Complications were particularly common in patients with type 2 diabetes. 8/14 patients (57%) with complications were patients with type 2 diabetes, while 8/11 patients (73%) with diabetes developed postoperative complications within the first 12 months. A positive correlation was shown between the features of type 2 diabetes mellitus and the occurrence of postoperative complications (r = .46; p = .005)). In our cohort, other systemic diseases such as hypertension, inflammatory dermatological diseases or rheumatic diseases did not show any significant correlation with development of postoperative complications.

Furthermore there was a significant difference in the development of complications between the two groups of patients with type 2 diabetes compared to patients without (t (34) = 3.02; p = .005).

## Discussion

In this study, we analyze clinical data from patients that received a penetrating corneal graft varying in size between 3.0 and 5.5 mm for corneal lesions that threatened the integrity of the globe. These lesions included ulcer and perforation, which were most commonly secondary to neurotrophic, inflammatory and infectious diseases.

Mini-keratoplasty is a treatment for small corneal ulcers with or without perforation. Trephination diameters of 3 to 5 mm can spare the host visual axis by using an eccentric small corneal graft. This technique has been advocated where non-surgical interventions, such as tissue adhesives, are considered insufficient, while larger grafts may result in an iatrogenic scar in the corneal center and are associated with a higher occurrence of immune rejection. [3], [6], [10]

Several authors have argued that mini-KP restores the integrity of the globe, while also allowing to completely excise the ulcerative lesion, alleviate inflammation and avoid complications, especially immune rejection [6], [10]. In cases with microbial keratitis, it can be used to reduce the risk of intraocular spread or scleral extension and supports epithelial healing, thus resulting in a reduced period of hospitalization and improved graft survival [11]. The immediate tectonic stability that keratoplasty ensures can improve the conditions for secondary, elective surgical interventions, if needed, to optimize visual function later.

In our cohort, only one out of 37 eyes required evisceration following a second corneal transplantation, while all other eyes were preserved by mini-KP. Another study showed similar results; all affected eyes were saved by small-diameter keratoplasty [6], [12]. In the patients described here, 23/37 or 62% of ees did not require further surgical treatment after mini-KP. When including patients who underwent only minor secondary corneal interventions but did not require a second graft, a total of 29 eyes (78%) acheved restoration of tectonic stability as a permanent solution.

At least 14/37 patients in our cohort had known autoimmune diseases, such as rheumatoid arthritis and inflammatory dermatological diseases. It is known that autoimmune diseases can have a negative impact on healing processes in human tissue. In the cornea, they can lead to new ulceration or graft rejection postoperatively and these complications are particularly frequent in eyes with neurotrophic keratopathy or rheumatoid arthritis [12]. In our cohort, diabetes mellitus type 2, compared to other systemic diseases, was a significant risk factor (t(34) = 3.02; p = .005) for post-operative complications requiring a second corneal intervention. Complications developed predominantly within the first four weeks after mini-KP. This suggests that close monitoring is required not only in patients with known autoimmune-diseases, but also diabetes mellitus, within the first weeks after mini-keratoplasty.

Previous studies have reported that keratoplasty for corneal ulceration related to herpetic keratitis carries a high risk of postoperative complications, especially recurrent infection and increased ocular surface inflammation, which potentially lead to graft rejection [1], [13], [14]. Ang et al. found that active corneal inflammation is associated with graft failure [1]. Also, patients affected by corneal ulceration related to herpetic keratitis were six times more likely to experience anatomic failure. These patients often have systemic autoimmune conditions with a known tendency for repeat melting due to the systemic nature of the inflammatory reaction [1]. However, in our study, all patients that suffered from ulcerative herpetic keratitis reached full anatomic success after mini-KP. Within our limited follow-up there were no graft rejections in this group. Hence, more research needs to be done regarding clinical outcomes after mini-keratoplasty in infectious keratitis, compared to regular-sized penetrating keratoplasty.

We defined mini-KP as a penetrating keratoplasty with a graft size of ≤ 5.5 mm diameter. Of course, graft size should be chosen as big as necessary and as small as possible [15–17]. Small diameter grafts result in less damage to the recipient cornea and require fewer corneal sutures, which can be an entry point for pathogens, cause intraocular infectionand lead to scar formation, potentially limiting functional outcome. Tischer et al. observed a higher frequency of immune reactions after penetrating keratoplasty with larger grafts and reduced distance between graft and corneoscleral limbus [10]. Higher ratios of graft size to recipient cornea size and small distance to the limbal margins in the Y-axis were significantly correlated with postoperative immune reactions [10]. Other studies have shown that smaller graft sizes, such as < 9.0 mm [1], < 8.0 mm [18]or < 6.0 mm [19], are significantly associated with lower risk of graft failure. Full-thickness grafts of a diameter less than 6 mm have shown better long-term survival rates than those of conventional size (7.0 to 8.0 mm) [19].

However, the eccentric position in which a mini-KP-graft is frequently placed means that one suture will be positioned closer to the optical center and the opposite suture will be closer to the limbus, [15], thus potentially limiting visual results due to irregular and higher-degree astigmatism, and increasing the risk of immune reaction due to more abundant immune cells and lymphatic vessels peripherally [10]. Due to lack of data regarding graft position and post-operative astigmatism in our retrospective study we could not analyze these interrelations.

Recent studies by Seifelnaser et al. and Roberts et al. have described a new technique of sutureless tectonic small diameter Descemet stripping automated endothelial keratoplasty (“mini-DSAEK”) for corneal perforations and investigated clinical outcomes highlighting intra- and postoperative surgical complications, astigmatism, and development of visual acuity in a total of six cases [20], [21]. Potential advantages outlined in those studies are avoidance of sutures and resulting complications, such as corneal vascularization [21]– [22]. Importantly, tectonic keratoplasty in peripheral perforation can result in surgically induced astigmatism and can cause significant visual impairment [20]. Also, the risk of immune reaction and subsequent graft failure may be reduced compared with a full thickness tectonic graft; However this has yet to be formally established. Sutureless DSAEK might be advantageous in small diameter, peripheral perforations in corneas with healthy endothelium regarding post-operative astigmatism and visual acuity. More research needs to be done to identify advantages of sutureless mini-DSAEK compared to full-thickness small diameter grafts.

In our study, we did not find significant visual improvement at six or twelve months. Scorcia et al. also reported poor visual outcomes after full-thickness grafts of small diameter, i.e., less than 6.0 mm [19], [23]. It is important to keep in mind that emergency penetrating mini-keratoplasty is an acute therapeutic intervention, which is performed to restore the integrity of the eye, eradicate acute infection and inflammation, and prevent further complications, while elective penetrating keratoplasty is often performed for optical reasons, if necessary, after mini-KP to achieve an improved optical clarity [3]. Other than that, there is the option to improve or restore visual acuity with a rigid gas permeable lens. This can sometimes lead to visual improvement without the need for a full thickness corneal transplant.

In our cohort best postoperative visual acuity of 0.4 dec (0.4 logMAR), which we consider sufficient for reading, was achieved in 10 of 36 eyes (28%) and independent outdoor orientation (equivalent to a CDVA of 0.05 dec (1.3 logMAR)), was achieved in 13 of 36 patients (36%). Thus 23/36 (64%) eyes at some point during the 12-months follow-up period had reliable visual acuity, although this was rather a secondary aim of the procedure [6]. Our study reports the outcome of mini-KP of ≤ 5.5 mm as an emergency treatment in patients with advanced corneal ulcers due to various infectious and noninfectious etiologies. All but one of the treated eyes were preserved after mini-keratoplasty. Over the 12-month followup most patients achieved stable results with moderate visual gains.

While this may be among the larger cohorts on this topic found in the literature and therefore warrant some statistical analysis, our findings are nonetheless limited by sample size and by the retrospective nature of our analysis. Results presented herein may form the basis for sample size calculation and study protocols of future, larger, prospective clinical investigations.

## Data Availability

The datasets used and/or analyzed during the current study available from the corresponding author on request.
